# Determination of Odor Compounds in Lignocellulose-Based Panels Using DHS-GC/MS Combined with Odor Activity Value Analysis

**DOI:** 10.3390/polym17172421

**Published:** 2025-09-06

**Authors:** Lina Tang, Qian Chen, Liming Zhu, Xiaorui Liu, Xianwu Zou, Yuejin Fu, Bo Liu

**Affiliations:** Research Institute of Wood Industry, Chinese Academy of Forestry, Beijing 100091, China; 18211090798@163.com (L.T.); chenqian0610@126.com (Q.C.); izhulm@caf.ac.cn (L.Z.); 18852003990@163.com (X.L.); xwzou@caf.ac.cn (X.Z.); bj-fyj@163.com (Y.F.)

**Keywords:** lignocellulose-based panels, odor compounds, dynamic headspace (DHS)–gas chromatography–mass spectrometry (GC-MS), odor activity value (OAV)

## Abstract

Wood, as the oldest natural polymer composite material on Earth, holds significant importance in the era of carbon neutrality and serves as an irreplaceable core material in the furniture and construction industries. As a primary raw material for furniture, wood-based lignocellulosic boards have drawn increasing consumer attention due to their odor characteristics. In order to achieve the determination of odor compounds in lignocellulose-based panels, this study established a method combining dynamic headspace sampling (DHS), gas chromatography–mass spectrometry (GC–MS), and odor activity value (OAV) analysis. To address the wide concentration range of odor compounds in lignocellulose-based panels, a three-level standard curve was established to meet the detection of odor substances in common lignocellulose-based panels. The favorable conditions for each factor were as follows: sheet-shaped samples, TENAX-TA adsorbent, 20 mL headspace vials, and a split ratio of 25:1. The method demonstrated good linearity within the range of 0.002–15 mg/m^3^, with recovery rates ranging from 94.74% to 103.44%. The method was applied to analyze commercially available particleboard, fiberboard, and plywood. A total of 33 odor components were detected. The results indicated that aldehyde contributed significantly to the odor of particleboard, acids were the main contributors to the odor of fiberboard, and terpenes dominated the odor of plywood. The established method is suitable for the qualitative and quantitative analysis of odor compounds in lignocellulose-based panels and provides reliable technical support for tracing, identifying, and controlling odors in these materials.

## 1. Introduction

Lignocellulose-based panels serve as primary raw materials in furniture, building materials, and other fields [1]. In recent years, with the enhancement of health awareness, consumers focus not only on harmful substances of lignocellulose-based panels such as formaldehyde, volatile organic compounds (VOCs), and heavy metals, but also on the increasingly prominent issue of odor [2]. At present, the comprehensive evaluation system and related standards for the odor of lignocellulose-based panels have been established [3], but there are still research gaps in the traceability analysis of their characteristic odor components. Identifying key odorants is essential to addressing odor problems in these materials. Researchers have used gas chromatography odor (GC-O) to detect odor components [4,5]. However, olfactory evaluation is inevitably influenced by subjective factors. Since odor perception depends on both concentration and odor threshold [6], in this paper, the main odor components were identified by calculating the odor activity value (OAV) [7], which has been widely used in multiple fields such as wine [8], tea [9], fruits [10], and flavorings [11].

Odors in lignocellulose-based panels mainly originate from volatile organic compounds. The traditional VOC collection method is based on an environmental chamber combined with thermal desorption gas chromatography/mass spectrometry (TD-GC/MS) [12]. This approach enables quantitative detection of VOCs in lignocellulose-based panels under controlled temperature, humidity, and air exchange rate. However, it is limited by high energy consumption and operational cost. Moreover, an air exchange rate of ≥0.5 h^−1^ may cause the escape of low-concentration compounds, increasing the risk of missing key components with high OAV. In contrast, in the dynamic headspace (DHS) method [13], an inert gas (e.g., nitrogen) is used to continuously purge the sample surface, thereby transferring and enriching the volatile odor compounds onto an adsorbent trap. This pretreatment offers high adsorption efficiency (>92%), excellent recovery of odor fingerprints, and full automation. It significantly reduces the loss of volatile odor components [14,15]. Therefore, this study developed a method based on TD-GC/MS for accurate qualitative and quantitative analysis combined with OAV screening to identify key odor compounds. The approach was applied to three major types of commercial lignocellulose-based panels (particleboard, fiberboard, and plywood) to analyze their characteristic odor components, which provides technical support for the identification of odor sources and production process optimization, thereby contributing to quality improvement in the industry.

## 2. Materials and Methods

### 2.1. Instruments and Reagents

The following instruments and reagents were used in this study: gas chromatography–mass spectrometry instrument (Agilent Corporation, Santa Clara, CA, USA, 8890-5977B), MPS-Robotic injector (Gestel, Germany), Tenax-TA tube (Gestel, Mülheim, Germany), electronic analytical balance (JJ500, 0.0001, Changshu Shuangjie G&G Measurement Plant, Changshou, China), widemouth bottle (Shanghai Bingyu Fluid Technology Co., Ltd., Shanghai, China), toluene (purity ≥ 99.8%), methanol (Shanghai Aladdin Company (Shanghai, China)).

### 2.2. Sample Preparation

The samples of lignocellulose-based panels were collected from the National Center of Inspection and Testing for Wood-based Panels and Wood-Bamboo Products. The main wood raw materials of the fiberboard used in the study of this article were mixed woods composed of hardwood (*Cinnamomum camphora* (L.) Presl, *Populus* sp., *Pterocarya stenoptera* C. DC, *Rhaphiolepis* sp., *Prunus* sp., *Fagus* sp., *Ligustrum* sp., *Firmiana* sp., *Melia* sp., *Salix* sp., *Celtis* sp., etc.) and a small amount of softwood (*Pinus* sp.).The main wood raw material of particleboard is a mixed wood composed of hardwood (*Eucalyptus* sp., *Melia* sp., *Bombax* sp., *Machilus* sp., *Bischofia* sp., *Saurauia* sp., *Celtis* sp., *Aleurites* sp., *Ficus* sp., *Bridelia* sp.) and a small amount of softwood (*Cunninghamiae* sp., *Pinus* sp., etc.). The wood raw material used in plywood was cypress wood. The particleboard, fiberboard, and plywood we tested only used urea formaldehyde resin throughout the entire production process; the resin content of particleboard and fiberboard was about 9% and 10%, respectively; and the glue spread of the plywood was 260 g/m^2^. The samples were sawn into three states: powder (40 mesh), sheet (8 mm × 5 mm × 1 mm), and block (8 mm × 5 mm × 25 mm).

### 2.3. Experimental Methods

#### 2.3.1. Methods of Dynamic Headspace

The sample (1.5 g) was placed into a 20 mL headspace vial, which was then hermetically sealed. DHS conditions were as follows: incubation at 60 °C for 30 min with shaking at 500 rpm; a capture volume of 1 L at a flow rate of 80 mL/min; an adsorption temperature of 30 °C for the trap; and a dry purge step with a volume of 30 mL delivered at 30 mL/min, during which the adsorption trap was maintained at 30 °C. For thermal desorption (TD3.5+), the parameters included a heating rate of 200 °C/min to a final temperature of 260 °C, which was held for 5 min, while the transfer line was also maintained at 260 °C. The cold injection system (CIS) was programmed with an initial temperature of −50 °C, followed by a heating rate of 10 °C/s to a final temperature of 260 °C, which was held for 5 min.

#### 2.3.2. Methods of GC-MS

Gas chromatographic separations were performed using a DM-TVOC quartz capillary chromatographic column (50 m × 0.32 mm × 1 μm). The injection port temperature was 250 °C. The gas flow rate was 1.53 mL/min. The temperature program was as follows: initial temperature 50 °C (held for 2 min), increased to 120 °C at 10 °C/min (held for 10 min), then to 160 °C at 5 °C/min (held for 10 min), followed by a ramp to 200 °C at 5 °C/min (held for 10 min), and finally to 250 °C at 5 °C/min (held for 5 min). For mass spectrometric detection, the interface temperature was maintained at 280 °C, the quadrupole at 150 °C, and the EI ion source at 230 °C. Electron impact ionization was performed at 70 eV, and mass spectra were acquired in the range of m/z 45–550.

#### 2.3.3. Data Analysis

Compounds were identified by comparing the mass spectra of peaks (S/N ≥ 10) with the INST database. Those with a similarity index greater than 80 (on a scale of 100) were selected for odor analysis. For the qualitative analysis, the mass spectrum of each unknown compound was first matched with the database, and the compound with the highest similarity was initially assigned. In cases of isomers or ambiguous identifications, elution order was considered—where compounds with lower boiling points typically elute earlier—as reflected by retention times. Characteristic fragment ions were also compared against the literature or database references. If uncertainty remained, confirmation was performed using authentic standard substances. Quantification was carried out using the external standard method. Given the similar properties between odor compounds and general volatile organic compounds, we followed the Chinese National Standard GB/T 29899-2024 [12], employing a toluene-based calibration curve for calculation. The calibration range for toluene was 0.0004–2.4 mg/mL.

#### 2.3.4. Analysis of Odor Substance Characteristics

Through systematic retrieval of cross-platform database resources such as PubMed, national and international standards, Flavornet, and Chemical Book, cross-validation and priority ranking of multi-source data of the target compound were conducted. The odor threshold and sensory characteristic description (olfactory profile) were integrated. The OAV of the substance was calculated according to Formula (1) [7]. Compounds with an OAV greater than 1 are generally considered to be key odorants.

(1) $${OAV}_{i}=\frac{C_{i}}{{OT}_{i}}$$

In the formula,

*OAV_i_*—odor activity value, dimensionless;

*C_i_*—the mass concentration of a certain volatile organic compound, with the unit of mg/m^3^;

*OT_i_*—the olfactory threshold of this volatile organic compound, with the unit of mg/m^3^.

### 2.4. Standard Solution Preparation and Volatilization Simulation

#### 2.4.1. Preparation of Mother Liquor

A toluene stock solution with a concentration of 3 mg/mL was prepared by dissolving 15 mg of toluene, weighed using an analytical balance with an accuracy of ±0.01 mg, in methanol within a 5 mL amber volumetric flask, which was then diluted to the mark.

#### 2.4.2. Preparation of Standard Working Solution

The standard working solution was obtained by stepwise dilution with methanol: 2.4, 2, 1, 0.6, 0.4, 0.2, 0.1, 0.04, 0.03, 0.012, 0.004, and 0.0004 mg/mL.

#### 2.4.3. Standardization of Filter Paper Load

Filter paper strips measuring 8 mm × 25 mm were used for the application of standard substances. All procedures were carried out in a climate-controlled chamber maintained at 25 ± 0.5 °C and 50 ± 2% relative humidity. A volume of 5 μL of the standard working solution was accurately delivered using a microsyringe and applied uniformly to the center of each filter paper strip. The prepared strip was immediately sealed in a headspace vial and allowed to equilibrate for 30 s before instrumental analysis. The calibration curve was plotted with the mass concentration of toluene as the abscissa and the peak area as the ordinate. 

## 3. Results and Discussion

### 3.1. Optimization of Instrument Conditions

#### 3.1.1. Optimization of Sample State

DHS technology enables the volatile and semi-volatile compounds in the sample to dynamically migrate into the headspace vial and be enriched in the adsorption tube through gradient temperature control and inert gas purging. The physical state of the sample will significantly affect the mass transfer efficiency. Therefore, in this paper, the sample states of powder, sheet, and block are discussed based on the size of the headspace vial and the sample dimensions.

**Figure 1:**
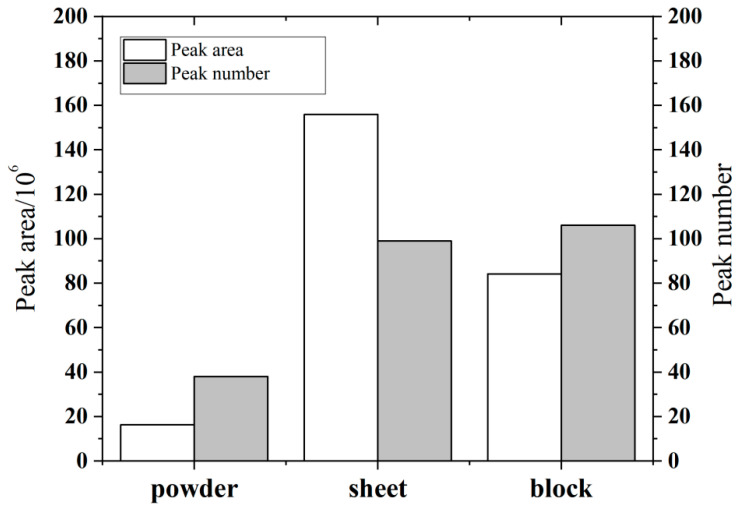
The peak area and number of peaks of powder, sheet, and block samples.

As shown in Figure 1, the powder sample exhibited the fewest detection peaks. This result may be attributed to its high specific surface area, which accelerates the escape of volatile components and leads to substantial losses during sample preparation. In contrast, both sheet samples and block demonstrated excellent detection responses. The former achieved more efficient substance release during thermal desorption by virtue of an appropriate geometric shape, while the latter effectively blocked component volatilization through a dense structure. Quantitative analysis indicated that the number of chromatographic peaks of sheet and block samples remained similar (38 ± 2 vs. 35 ± 3), but the total peak area of sheet samples showed a significant difference compared with block samples ($1.6\times 10^{8}\;vs.\;0.8\times 10^{8}$), and its response intensity reached 1.9 times that of the latter. This phenomenon may be related to the unique surface volume ratio advantage of the sheet-like morphology. That is, under the same mass conditions, the sheet-like structure can not only maintain the integrity of the composition through planar extension, but also form gradient heat conduction through the unilateral heating mode, thereby achieving more complete compound release during the thermal desorption process. Based on the dual optimization of the retention efficiency and release efficacy of volatile components, the sheet form was selected as the optimal sample configuration for this study.

#### 3.1.2. Optimization of Headspace Vial Volume

**Figure 2:**
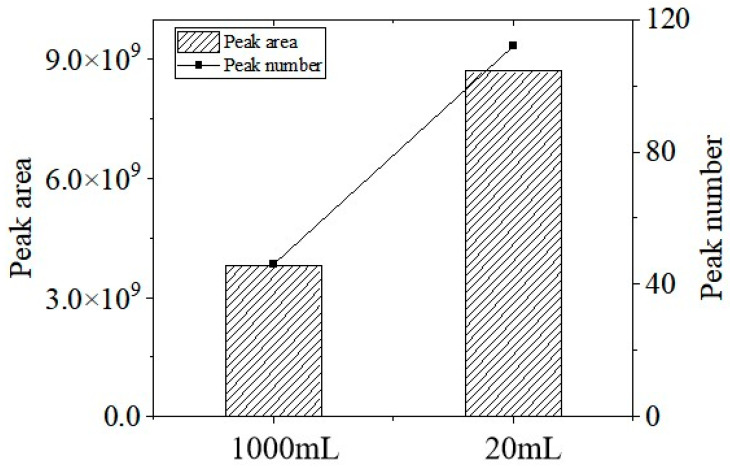
Total ion flow diagram affected by headspace bottle volume.

As shown in Figure 2, a comparative screening experiment was conducted using 1 L large-volume headspace vials and 20 mL small-volume headspace vials. The results demonstrate a marked improvement in performance with the smaller vials: the total chromatographic peak area increased approximately three-fold, while the number of detected peaks rose by roughly two-fold compared to the large-volume vials. This enhancement can be attributed to more efficient air circulation and displacement within the smaller vial volume when a consistent purge flow rate of 100 mL/min is used. The improved fluid dynamics promote more thorough gas–solid mass transfer of volatile compounds, leading to higher recovery rates and better detection sensitivity. Based on these findings, a 20 mL headspace vial was used for analysis in this study.

#### 3.1.3. Selection of Packing for Adsorption Tubes

**Figure 3:**
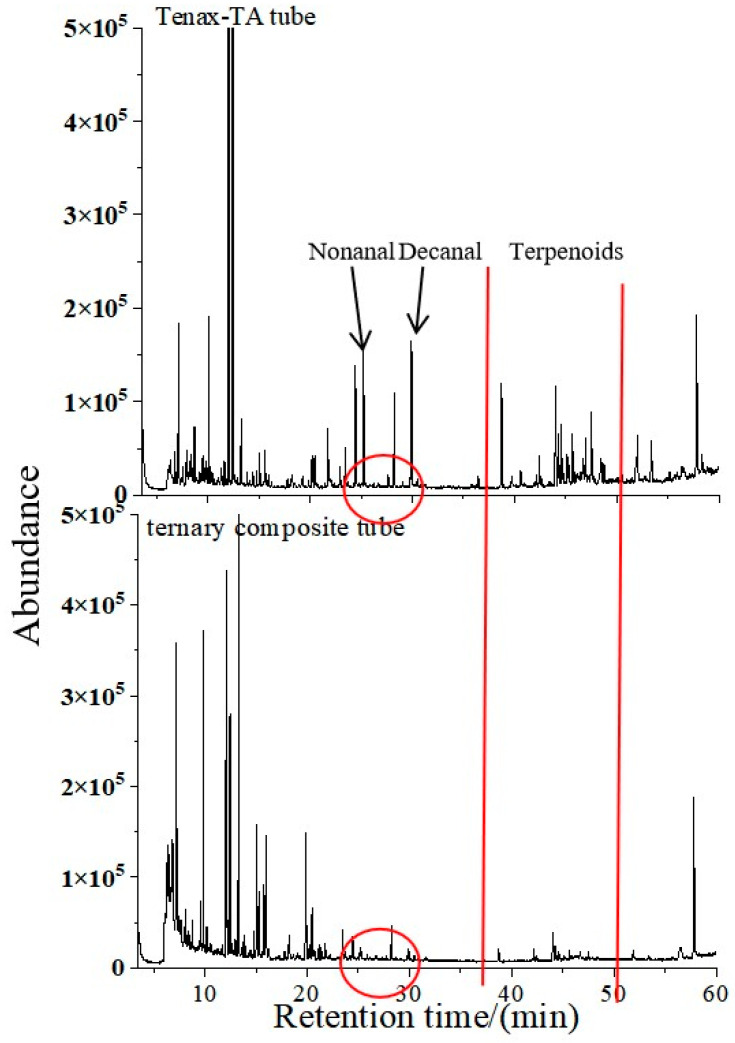
Comparison of total ion flow diagrams between two adsorption tubes.

As shown in Figure 3, there was a significant difference in adsorption selectivity between the Tenax-TA single-packed tube and the ternary composite tube (Tenax-GR/microporous carbon/Carboxen1000). Overall, the Tenax-TA tube demonstrated superior adsorption performance compared to the composite tube. In the low-temperature desorption stage (retention time: 5–25 min), the adsorption capacity of the two adsorption tubes for VOCs was comparable. This might be attributed to the synergistic trapping effect formed by the microporous structure (pore size 0.8–1.2 nm) of the Carboxen1000 in the composite tube and the gradient pore size distribution (1–50 nm) of the medium and microporous carbon. However, the composite tube exhibited noticeably diminished performance during the high-temperature desorption stage. For instance, between 25 and 30 min, compounds such as nonanal and decanal were detected in the Tenax-TA tube but were absent in the composite tube. Furthermore, in the retention interval of 38–50 min, where terpenoids (e.g., α-pinene and β-pinene) were the major detected compounds, the adsorption efficiency of the composite tube decreased sharply, falling below the detection limit. Therefore, the Tenax-TA tube was selected as the primary adsorbent in this study. The combined tube can be used as a supplementary adsorption device for C5–C12 compounds, and the full-component analysis of VOCs can be achieved through the dual-tube combination strategy.

#### 3.1.4. Optimization of the Temperature Rise Procedure of the Column Oven

**Figure 4:**
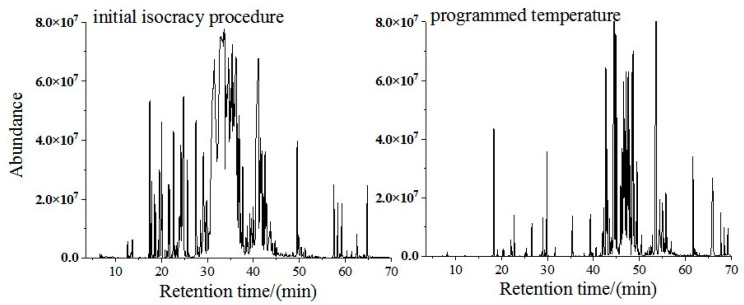
Total ion flow diagram compared by temperature-programmed method.

The optimization of the column oven temperature program is shown in Figure 4. The initial isocratic procedure (maintained at 50 °C for 5 min and increased from 10 °C/min to 250 °C) resulted in severe co-elution of C14–C16 volatile components during 30–45 min. Critical compounds such as longibrene, β-caryophyllene, cedar alcohol, and cedarene exhibited resolutions ≤0.75, which is significantly below the required minimum of 1.5 for adequate separation. A four-step temperature gradient was therefore optimized as follows: ① 50 °C (2 min) →② 10 °C/min to 120 °C (10 min) → ③ 5 °C/min to 160 °C (10 min) → ④ 5 °C/min to 200 °C (10 min) → ⑤ 5 °C/min to 250 °C (5 min). This optimized program markedly improved chromatographic performance: the total number of detected peaks increased from 72 ± 5 to 135 ± 8, and the signal-to-noise ratio of key compounds improved by a factor of 4.3.

#### 3.1.5. Optimization of the Split Ratio

**Figure 5:**
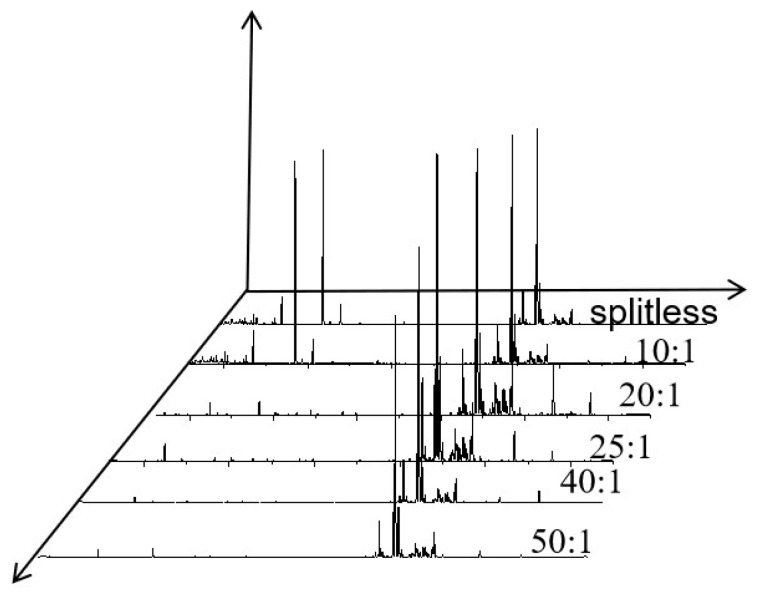
Total ion flow diagram influenced by shunt ratio.

**Figure 6:**
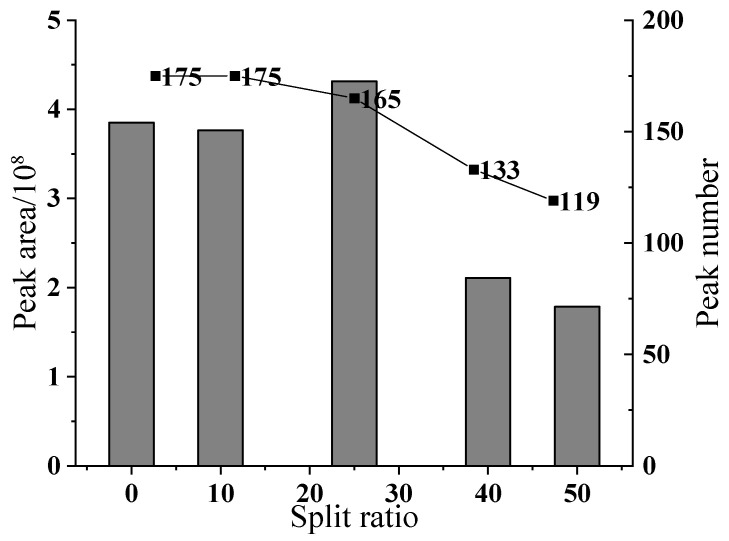
Relationship diagram of shunt ratio, peak area, and peak number.

As demonstrated by the split ratio optimization experiment (Figure 5), a distinct nonlinear relationship was observed between injection volume adjustment and chromatographic outcomes. When the split ratio increased from splitless to 50:1, quantitative analysis (Figure 6) revealed that the split ratio of 25:1 achieved the optimal balance in multiple indicators. At this ratio, the total peak area reached the highest, increasing by approximately 1.1 times compared to the splitless. Further increasing the split ratio resulted in a sharp decline in both the total peak area and the number of detectable peaks. Moreover, the resolution of key compounds such as α-cedarene increased from 1.43 to 1.52 (R ≥ 1.5). This improvement can likely be attributed to the relatively high column loading capacity and favorable distribution coefficient achieved under the 25:1 split ratio.

### 3.2. Methodological Validation

#### 3.2.1. Linear Range and Detection Limit

**Figure 7:**
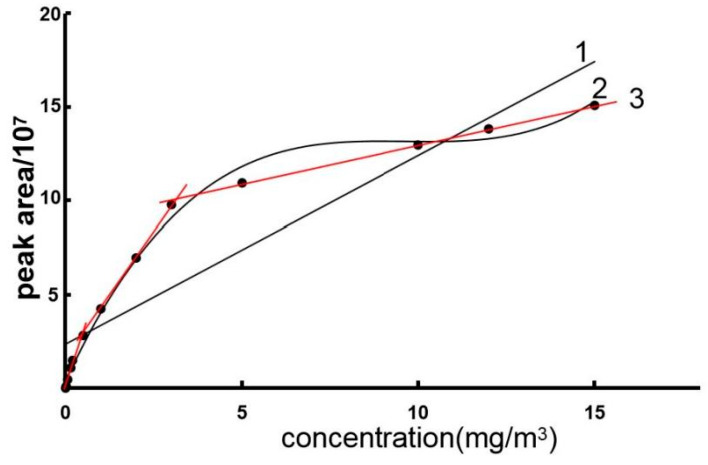
Fitting of toluene standard curves (1 is the curve fit by a traditional continuous regression model, 2 is the curve fit by a traditional polynomial model, 3 is the piecewise linear regression model).

The construction results of the standard curves are shown in Figure 7. Curve 1 was obtained by a conventional continuous regression model, while curve 2 corresponds to a traditional polynomial model. As illustrated, both models exhibited unsatisfactory fitting performance, with correlation coefficients of R^2^ = 0.838 and R^2^ = 0.994, respectively, failing to meet experimental requirements.

**Figure 8:**
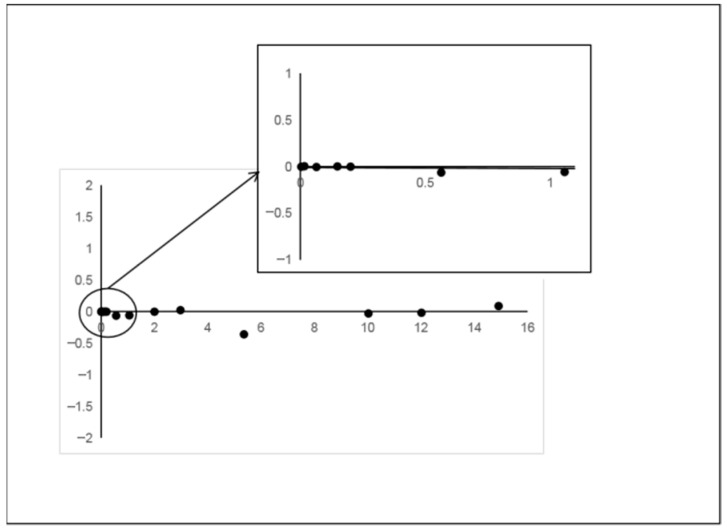
Residual plot of segmented calibration curve.

The underlying causes may be attributed primarily to two factors: First, the capillary permeation effect of the filter paper fibers leads to a certain deviation between the droplet diffusion area and the theoretical value. Second, temperature fluctuations cause uneven evaporation rates. For this purpose, a concentration-dependent piecewise linear regression model (Table 1) was introduced. The detection range was divided into three kinetic domains: low-concentration domain (0.002–0.2 mg/m^3^), medium-concentration range (0.2–3 mg/m^3^), and high-concentration domain (3–15 mg/m^3^). Residual analysis (Figure 8) showed that the residuals were randomly distributed up and down within ±0.3 around the *X*-axis, indicating a good fit [16].

#### 3.2.2. Recovery Rate and Precision of Spiked Products

The results of the spiked recovery experiment carried out in the lignocellulose-based panel matrix (with a spiked amount of 2 mg/m^3^, *n* = 5) are shown in Table 1. Method validation demonstrated a recovery range of 94.74–103.44%, with a relative standard deviation of 3.2%, confirming that the optimized method exhibits good accuracy and precision.

### 3.3. Sample Determination

The odor compounds in three types of commercially available lignocellulose-based panels (particleboard, fiberboard, and plywood) were determined by dynamic headspace gas chromatography–mass spectrometry (DHS-GC/MS) combined with OAV analysis. The results are shown in Table 2. A total of 33 characteristic odor compounds were identified, mainly including aldehydes (11 kinds), acids (4 kinds), terpenoids (9 kinds), monoaromatic series (3 kinds), ketones (2 kinds), alcohol (1 kind), ester (1 kind), halohydrocarbon (1 kind), and ether (1 kind). Aldehydes were the most diverse group. Their odors were grassy and food source (such as peach, apple, and oils) odors, with the exception of isobutyraldehyde, which presented a pungent odor. Among acids, from acetic acid to butyric acid, the odor transitioned from sharp and irritating to mild as the carbon chain lengthened; benzoic acid even emitted a faintly fragrant scent. Terpenoids mainly had a woody or floral smell, while monoaromatic compounds contributed aromatic qualities. Alkanes and alcohols had the odor of chloroform or ether. Eerolidyl acetate had a fresh woody aroma. Acetone had a pungent and irritating smell, while 1-hydroxy-2-Propanone had a caramel odor.

A total of 15 odor compounds were identified in the particleboard. Key odor compounds included hexanal (OAV = 322.869), nonanal (OAV = 271.419), acetic acid (OAV = 125.786), 3-methyl-butyraldehyde (OAV = 92.122), heptaldehyde (OAV = 40.762), valeraldehyde (OAV = 20.954), isobutyraldehyde (OAV = 10.573), and benzaldehyde (OAV = 3.085). Aldehydes constituted the highest proportion at 60% (total OAV = 762.4) and were the primary contributors to the odor profile of particleboard. This result was consistent with the research results of Liu [2] and Tian [17]. The main reasons were as follows: First, both the wood chips and adhesives used in particleboard production release aldehydes [18]. Second, aldehyde compounds exhibit a relatively low odor threshold.

A total of nine odor compounds were identified in the fiberboard. Key odor compounds included butyric acid (OAV = 9.326), acetic acid (OAV = 8.069), heptanal (OAV = 3.867), and hexanal (OAV = 2.798). Acids accounted for approximately 50% of the total odor contribution (total OAV = 22.1), identifying them as the main odor sources in fiberboard. This was consistent with what Wang reported [19].

Thirteen odor compounds were identified in plywood, with terpenoids being the most dominant group, comprising 61.5% of the key odorants. Major terpenoids included α-bisabolol (OAV = 105.638), longifolene (OAV = 45.135), α-cedrene (OAV = 36.973), cedrol (OAV = 24.593), beta-acorenol (OAV = 3.497), cis-thujopsene (OAV = 2.573), and alpha-terpineol (OAV = 1.150). Other notable compounds were hexanal (OAV = 40.079), nonanal (OAV = 30.813), and 1-methoxy-2-propanol (OAV = 4.899). The total OAV reached 219.6. Terpenoids contributed the most to the overall odor of plywood. Meanwhile, both the diversity and concentration of terpenoids in plywood were markedly higher than those in particleboard (1 type) and fiberboard (0 types). Terpenoids mainly originate from wood itself. Therefore, the type of wood substrate is the core factor influencing the types and contents of terpenoids in lignocellulose-based panels. The volatile components of camphor wood and pine wood are generally greater than those of poplar and fast-growing eucalyptus [20]. Thus, the content of terpenoids in lignocellulose-based panels made of camphor wood and pine wood is relatively high. Furthermore, plywood is manufactured using rotary-cut or sliced veneers [21], which better preserve the natural anatomical structures of wood, such as resin canals, compared to shredded fibers or particles. This structural integrity helps retain more extractives, including terpenoids. Additionally, the presence of resin channels reduces the loss of these compounds during hot pressing, resulting in higher terpenoid retention in plywood.

Additionally, 2-methylbutyraldehyde has been reported as the material basis for the generation of “aroma” during malt roasting [22]. It is also a fragrance, insect repellent, and organic synthesis intermediate [23]. Currently, the odor threshold of furfural has only been documented in water, at 282 ug/L [24]. Although the threshold is relatively high, during the manufacturing process of lignocellulose-based panels, especially fiberboards, under high-temperature hot pressing, the hemicellulose or free sugars in the wood are hydrolyzed and converted into furfural [25]. Benzoic acid and 1, 3-dichloro-2-propanol may originate from adhesives, and widdrol and (-)-camphene may originate from wood. Therefore, the odor thresholds of these compounds in the air deserve further determination to obtain a more accurate odor evaluation.

**Table 2:** Concentration and odor activity values of odor compounds in lignocellulose-based panels.

Compounds		Retention Time (min)	Characteristic Fragment Ions (*m*/*z*)	Odor	Odor Threshold mg/m^3^	Particleboard		Fiberboard		Plywood	
						Concentration mg/m^3^	OAV	Concentration mg/m^3^	OAV	Concentration mg/m^3^	OAV
Aldehydes	isobutyraldehyde	8.95	72, 57, 73, 55	A pungent odor *	0.0010 *	0.011	10.573	-	-	-	-
	methacrolein	9.21	70, 69, 71	Wild hyacinth scent *	0.0244 *	0.018	0.721	-	-	-	-
	isovaleraldehyde	11.12	58, 57, 71	Ethereal, chocolate, and peach flavors *	0.0004 *	0.032	92.122	-	-	-	-
	2-methylbutyraldehyde	11.38	57, 58, 43, 86	Moldy smell, fermented baking smell *	-	0.015	-	-	-	-	-
	pentanal	12.09	58, 57, 71, 86	Grassy [26]	0.0014 [27]	0.029	20.954	-	-	-	-
	hexanal	14.84	56, 57, 72	Stimulating grassy scent and apple aroma [26]	0.0011 [26]	0.355	322.869	0.003	2.798	0.044	40.079
	furfural	17.44	96, 95, 67	Sweet woody almond-flavored toasted bread mixed with aroma [24]	-	-	-	0.006		-	-
	heptanal	18.26	70, 55, 57	fruity aroma [26]	0.0008 [26]	0.033	40.762	0.003	3.867	-	-
	benzaldehyde	20.97	77, 106, 105	Bitter almond flavor [26]	0.0260 [26]	0.080	3.085	-	-	-	-
	nonanal	30.38	57, 56, 55, 70	Strong oily and sweet orange aroma [26]	0.0020 [26]	0.543	271.419	-	-	0.062	30.813
Acids	acetic acid	5.93	45, 60	Strong, pungent, vinegar-like odor *	0.0147 *	1.854	125.786	0.119	8.069	-	-
	propanoic acid	10.24	74, 45, 73	The taste of spicy acidic cheese vinegar *	0.0173 *	-	-	0.015	0.880	-	-
	butanoic acid	15.60	60, 73, 45	Yogurt and cheese flavor *	0.0007*	-	-	0.006	9.326	-	-
	benzoic acid	31.29	105, 122, 77	Light fragrance *	-	-	-	0.003		-	-
Terpenoids	alpha-terpineol	29.85	59, 93, 122, 136	Lilac aroma [26]	0.3855 [26]	-	-	-	-	0.443	1.150
	longifolene	44.08	161, 94, 91, 107	Wood fragrance and iris-like aroma [26]	0.0024 [26]	-	-	-	-	0.108	45.135
	α-cedrene	44.31	119, 93, 105	Sweet and gentle cypress characteristic aroma [27]	0.0113 [26]	-	-	-	-	0.418	36.973
	cis-thujopsene	45.02	119, 123, 105, 121	A faint woody fragrance [26]	0.0630 [26]	-	-	-	-	0.162	2.573
	widdrol	53.12	151, 95, 69	Odorous [28]	-	-	-	-	-	0.474	-
	cedrol	53.44	95, 150, 151	Weak wood fragrance with some ointment fragrance [26]	0.5126 [26]	-	-	-	-	12.607	24.593
	β-acorenol	54.62	119, 121, 161	Woody [26]	0.0144 [26]	-	-	-	-	0.050	3.497
	α-bisabolol	56.87	69, 109, 119	Woody [26]	0.0013 [26]	-	-	-	-	0.137	105.638
	(-)-camphene	43.61	93, 121, 79	Fir terpene flavor *	-	0.053	-	-	-	-	-
Monoaromatic series	benzene	11.60	78, 77, 519	Aromatic odor [26]	8.6130 [26]	0.010	0.001	-	-	-	-
	toluene	14.36	91, 92, 65	Special fragrant aroma [26]	1.2408 [26]	0.037	0.030	-	-	-	-
	p-xylene	17.69	91, 106, 105	Stimulating aromatic scent [26]	0.2517 [26]	0.026	0.103	-	-	-	-
Alcohol	1,3-dichloro-2-Propanol	15.21	79, 81, 49	Ether odor *	-	-	-	-	-	0.160	
Ester	nerolidyl acetate	45.70	69, 93, 107	Fresh, sweet, citrus and orchid wood fragrance *		0.058	-	-	-	-	-
Ketones	1-hydroxy-2-propanone	7.84	74, 45	Caramel aroma *	-	-	-	0.007	-	-	-
	acetone	7.16	58, 59, 57	Spicy and stimulating [18]	99.76 [29]	-	-	-	-	0.447	0.005
Halohydrocarbon	1,2-dichloro-ethane	7.68	62, 64, 49, 63	Chloroform-like odor	-	-	-	0.004			
Ether	1-methoxy-2-propanol	9.87	45, 47, 75	Sweet ether-like odor *	0.012 [25]	-	-	-	-	0.060	5.000

Note: * indicates that the data is sourced from the Chemical Book website. - indicates that no result was found or the result cannot be calculated.

## 4. Conclusions

An optimized analytical method was established for odor substances in lignocellulose-based panels characterized by dynamic headspace extraction, gas chromatography–tandem mass spectrometry analysis, and odor activity value (OAV) characterization. The method demonstrated a wide linear range of 0.002–15 mg/m^3^, a detection limit of 0.0005 mg/m3, spiked recovery rates between 94.74 and 103.44%, and an RSD value of 3.2%. These results indicate high sensitivity, good reproducibility, and a broad linear response, confirming the reliability of the method for detecting characteristic odor compounds in commercial particleboard, fiberboard, and plywood.

A total of 33 compounds were identified, including aldehydes, acids, terpenoids, monoaromatic series, ketones, alcohol, ester, halohydrocarbon, and ether. Among them, aldehydes were found to be the major odor contributors in particleboard, acids in fiberboard, and terpenoids in plywood. The method established in this study enables accurate quantification of odor compounds in lignocellulose-based panels and facilitates the identification of key odorants, providing technical support for the odor traceability and treatment of lignocellulose-based panels.

## Figures and Tables

**Table 1:** Recovery rate of labeled plywood samples.

No.	Original (mg/m^3^)	Added (mg/m^3^)	Detected (mg/m^3^)	Recovery (%)	RSD (%)
1	0.43	2.00	2.32	94.7	3.2
2	0.44	2.00	2.50	103.2	
3	0.45	2.00	2.51	103.0	
4	0.43	2.00	2.50	103.4	
5	0.43	2.00	2.46	101.6	

## Data Availability

The raw data supporting the conclusions of this article will be made available by the authors on request.
